# Clinical Utility of Machine-Learning Approaches in Schizophrenia: Improving Diagnostic Confidence for Translational Neuroimaging

**DOI:** 10.3389/fpsyt.2013.00095

**Published:** 2013-08-29

**Authors:** Sarina J. Iwabuchi, Peter F. Liddle, Lena Palaniyappan

**Affiliations:** ^1^Division of Psychiatry, Centre for Translational Neuroimaging in Mental Health, University of Nottingham, Nottingham, UK; ^2^Nottinghamshire Healthcare NHS Trust, Nottingham, UK

**Keywords:** gray matter, machine learning, schizophrenia, structural MRI, support vector machine, white matter, diagnosis

## Abstract

Machine-learning approaches are becoming commonplace in the neuroimaging literature as potential diagnostic and prognostic tools for the study of clinical populations. However, very few studies provide clinically informative measures to aid in decision-making and resource allocation. Head-to-head comparison of neuroimaging-based multivariate classifiers is an essential first step to promote translation of these tools to clinical practice. We systematically evaluated the classifier performance using back-to-back structural MRI in two field strengths (3- and 7-T) to discriminate patients with schizophrenia (*n* = 19) from healthy controls (*n* = 20). Gray matter (GM) and white matter images were used as inputs into a support vector machine to classify patients and control subjects. Seven Tesla classifiers outperformed the 3-T classifiers with accuracy reaching as high as 77% for the 7-T GM classifier compared to 66.6% for the 3-T GM classifier. Furthermore, diagnostic odds ratio (a measure that is not affected by variations in sample characteristics) and number needed to predict (a measure based on Bayesian certainty of a test result) indicated superior performance of the 7-T classifiers, whereby for each correct diagnosis made, the number of patients that need to be examined using the 7-T GM classifier was one less than the number that need to be examined if a different classifier was used. Using a hypothetical example, we highlight how these findings could have significant implications for clinical decision-making. We encourage the reporting of measures proposed here in future studies utilizing machine-learning approaches. This will not only promote the search for an optimum diagnostic tool but also aid in the translation of neuroimaging to clinical use.

## Introduction

Several years of neuroimaging research has established a convincing association between psychotic disorders such as schizophrenia and the presence of structural and functional alterations in the brain. Of late, the advent of refined statistical approaches such as machine-learning algorithms for pattern classification has provided a means to use neuroimaging as a clinical diagnostic or prognostic tool. Consequently, neuroimaging has been brought under the spotlight of translational research in psychosis. Systematic estimation of the clinical utility of various neuroimaging tools is a relevant necessity, more than ever before.

Conventionally, a diagnosis for a psychotic disorder relies on information gathered during clinical interviews. However, a number of neuroimaging studies using machine-learning approach on both functional (1–6) and structural (3, 4, 7–23) data, have attempted to provide diagnostic information applicable to individual patients albeit with varying degrees of success. Pattern classification methods used in these studies differ from the more conventional imaging methods that use general linear models [e.g., voxel based morphometry (VBM)] by utilizing a multivariate approach to identify discriminating features. Mass univariate analyses such as VBM look for differences in localized regions of the brain that are extracted from group differences. Pattern classification on the other hand, seeks out subtle differences in patterns across the brain that best discriminates diagnostic groups. As a result, univariate approaches often yield effects sizes that are too small to allow useful conclusions to be drawn in individual cases, whereas multivariate analyses offer a greater prospect of diagnosing (i.e., correctly identifying the group membership) individual cases.

A validated set of multivariate features (classifier) that provides the best discrimination between two diagnostic groups can be applied to a “new” individual to identify the diagnostic category to which he/she may belong. This applicability at the level of the single individual makes pattern classification approach a potentially valuable diagnostic and/or prognostic tool.

A major challenge with the wider use of machine-learning approaches is the lack of consistency in test performance (measured using Accuracy, Specificity, and Sensitivity) even across studies distinguishing patients from healthy controls, a discrimination that is often reliably done in clinical practice. In part this can be attributed to the varied selection of neuroimaging modalities [resting state or task based functional MRI, white matter (WM) or gray matter (GM) morphometry, diffusion tensor imaging] in classification studies. But discrepancies are observed even amongst studies using the same imaging modality. For example, Borgwardt et al. (8) and Pettersson-Yeo et al. (4) used highly similar patient groups with similar sample size and employed a non-linear support vector machine (SVM) for a GM classifier. However, results were very different; Borgwardt et al.’s (8) classifiers separated controls and first-episode psychosis patients with a high rate of accuracy (86.7%), while Pettersson-Yeo et al. (4) study failed to discriminate these two groups. Inconsistencies such as these may be due to a variety of factors such as severity of disease in patient groups, and image acquisition specifications (e.g., varying scanner strengths, scanning durations). Higher variation in performance has been previously noted when different field strengths (1.5 vs. 3 T) were used to classify patients with Alzheimer’s dementia from healthy controls (24). In addition, the most commonly reported test performance measure Diagnostic Accuracy is prone to vary with prevalence of the disease in the study population (25). For neuroimaging to be promoted as an aspect of routine clinical care in psychosis, it is important to compare various modalities and data acquisition strategies preferably using measures that are not affected by sample characteristics and establish the best performing methods. In addition, to enable clinicians to utilize these tools in the “real-world” of clinical practice, it is important that we report appropriate indices that emphasize the *clinical significance* of various classification approaches.

The current study aims to explore whether SVM classifiers intended to distinguish patients with schizophrenia from healthy controls perform differently when based on two modalities (GM and WM morphometry) of structural magnetic resonance imaging (sMRI) data acquired in a back-to-back fashion in the same sample at two different scanner strengths (3 and 7 T MR scanners). Given the superior signal-to-noise ratio (SNR) for 7 T MRI, and the predominance of GM changes in schizophrenia, we expected 7 T and GM based classifiers to outperform 3 T and WM based classifiers. In addition to demonstrating statistically significant changes among the classifiers, our major interest was to estimate the extent of any incremental gain in a clinically meaningful manner. To this end, for the first time in pattern classification studies we have estimated the number of patients required to be tested in order to correctly predict diagnosis in one person (number needed to predict, NNP) using a Bayesian measure of diagnostic certainty (26). Further, in addition to sensitivity, specificity, and diagnostic accuracy, we report diagnostic odds ratio (DOR), a composite indicator of test performance that summarizes sensitivity and specificity without being affected by variations in disease prevalence (27).

## Materials and Methods

### Participants

Twenty patients with schizophrenia and 21 healthy controls were recruited for the study, of which 19 patients and 20 healthy controls with scans of adequate quality are included in the current analyses. Patients were aged between 18 and 55 years and were diagnosed with schizophrenia according to the DSM-IV. A consensus procedure after reviewing clinical notes, collecting information from the psychiatrists providing clinical care and a structured clinical interview [Signs and Symptoms in Psychotic Illness (28)] was conducted with each patient. All patients were in a stable phase of illness [defined as a change of no more than 10 points in their Global Assessment of Function (GAF, DSM-IV) score, assessed 6 weeks prior and immediately prior to study participation]. The mean duration of illness was 7.7 years (SD = 8.3). Subjects with age<18 or>55, subjects with neurological disorders, current substance dependence, or IQ < 70 using Quick Test (29) were excluded. Healthy controls group-matched for age, gender, and parental socio-economic status with the patients were recruited from the local communities through advertisements. In addition to the exclusion criteria specified for patients, controls were excluded if there was a personal or family history of psychosis. All subjects were recruited from Nottinghamshire, UK. Permission for the study was obtained from National Research Ethics Committee, Nottingham, UK. All participants gave written informed consent.

### MRI data acquisition

Scanning was performed on a 3 and 7-T Philips Achieva system with 32-channel receive coil. Three Tesla magnetization-prepared rapid acquisition gradient echo (MPRAGE) images were obtained with 1 mm isotropic resolution, 256 × 256 × 160 matrix, Repetition Time (TR) = 8.1 ms, Echo Time (TE) = 3.7 ms, shot interval 3 s, flip angle 8°was acquired for each participant. Seven Tesla T1 weighted images were acquired using a 3D Magnetization Prepared – Turbo Field Echo (IR-TFE) with 0.6 mm isotropic resolution, 192 mm × 180 mm × 140 mm matrix, TR = 15 ms, TE = 5.6 ms, shot interval = 3 s, flip angle 8°. An optimized inversion pulse (adiabatic pulse) was used at 7 T to reduce bias field inhomogeneity. The 3- and 7-T scans were acquired one after the other in the same order (3 T followed by 7 T) on the same day for all subjects, with 5–10 min of time interval between the scans for transferring the patients between two scanners located in the same building. One patient and one control were excluded due to significant movement artifacts that precluded analysis. Clinical and demographic features of the sample are presented in Table 1.

**Table 1:** **Clinical and demographic features**.

Features	Patients (*N* = 19) mean (SD)	Controls (*N* = 20) mean (SD)
Gender (male/female)	14/5	15/5
Handedness (right/left)	17/2	18/2
Age	33.2 (9.8)	32 (8.2)
Parental NS-SEC	2.6 (1.7)	2.5 (1.6)
SSPI total score*	11.1 (10)	0.6 (0.8)
Reality distortion*	2.4 (2.9)	0 (0)
Disorganization*	0.2 (0.7)	0.1 (0.3)
Psychomotor poverty*	2.3 (3.7)	0 (0)
Illness duration	7.7 (8.3)	–
DDD of antipsychotics	0.8 (0.7)	–
GAF score*	47.3 (10.7)	88.1 (7.4)

SD, standard deviation; NS-SEC, national statistics socio-economic classification; SSPI, signs and symptoms of psychotic illness; DDD, defined daily dose; GAF, global assessment of functioning. *Significantly different between the two groups using Mann–Whitney U test (p < 0.05).

### Image processing and SVM classification

**Figure 1:**
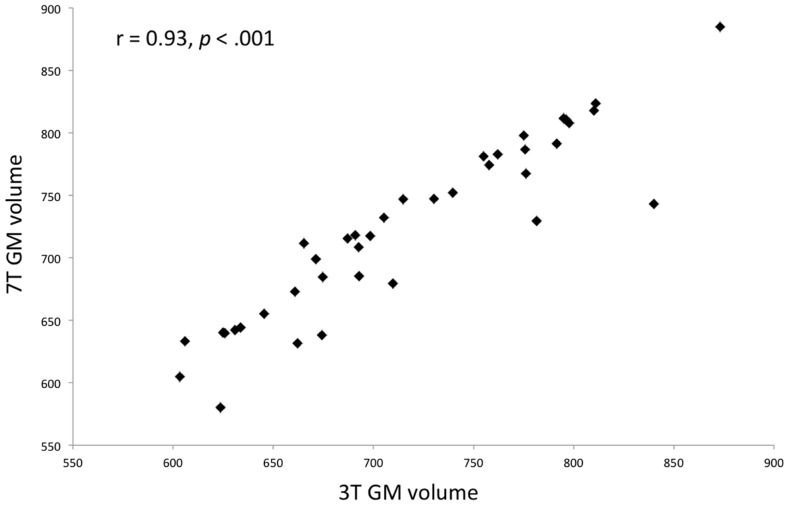
**Scatterplot showing a strong correlation between 3 and 7 T gray matter volumes after segmentation using SPM8 DARTEL algorithm for the entire sample (both patients and controls)**.

T1 images were resliced (1 mm isotropic) and segmented into gray, white, and CSF tissue using the SPM8 Diffeomorphic Anatomical Registration Through Exponentiated Lie algebra (DARTEL) algorithm (30). GM and WM images were separately warped onto a group average template and normalized to MNI space. To correct for variation due to field inhomogeneity, the images were bias field corrected using 60 mm FWHM setting using SPM8 (31). To confirm that the higher inhomogeneity in the ultra-high field (7 T) did not affect the integrity of tissue segmentation process, we compared the total GM tissue volume from the 3- to 7-T scans. There was no significant difference [paired *t*(38) = 0.18, *p* = 0.9] in the GM volume. Furthermore, the total GM tissue volumes obtained from 3 to 7 T scans were very highly correlated (*r* = 0.93, *p* < 0.001), indicating that there were no systematic differences in the tissue segmentation between the 3- and 7-T scans (Figure 1). The normalized, modulated, unsmoothed WM, and GM images for the 3- and 7-T datasets were then used as inputs to the separate linear SVM classifiers. In this approach, each subject’s input image is considered as a datapoint in a high-dimensional space of anatomical information (defined by GM or WM volumes). A hyperplane producing the greatest margin between the datapoints of the opposite groups (controls and patients) was identified using the multivariate information from the input images. A linear rather than non-linear kernel matrix was computed as input into the SVM classifier, as this allows the extraction of weight vectors of the high-dimensional data and also reduces the likelihood of overfitting (32). The SVM analysis was carried out using Pattern Recognition for Neuroimaging Toolbox (PRoNTo), following the standard manualized descriptions (http://www.mlnl.cs.ucl.ac.uk/pronto).

Kernel-based approaches such as the one used here utilize a similarity matrix derived from all datapoints when developing classifiers; this obviates the need for explicit dimensionality reduction and optimizes computation efficiency (33). To measure the test performance and to validate the classifier, a leave-one-subject-out (LOSO) cross validation approach was employed, where the classifier is trained on all subjects except one, which is used as test data. Balanced accuracy, specificity, sensitivity, and predictive values for each classifier were obtained and statistical significance of these measures was determined by way of permutation testing (*n* = 1000 permutations with random assignment of patient/control labels to the training data).

### Discrimination maps

**Figure 2:**
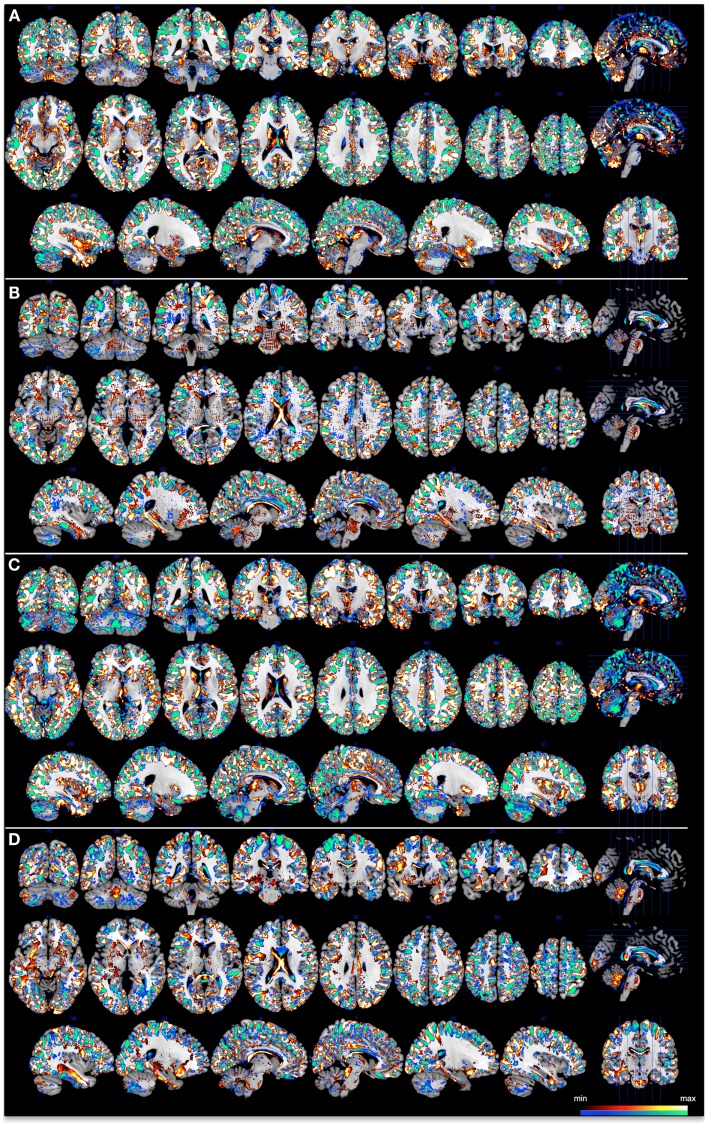
**Discrimination maps for each classifier at a threshold of 30% of the maximum positive and negative weight values, superimposed onto a standard brain template provided by MRICron**. **(A)** 3 T GM, **(B)** 3 T WM, **(C)** 7 T GM, and **(D)** 7 T WM. Color bar represents the minimum and maximum thresholded weights for each classifier.

Each voxel carries a certain weight value signifying its contribution toward the classification function. This value can be positive or negative, where a positive value would represent a higher weighted average for class one (controls group), while a negative value would mean the weighted average was higher for class two (patient group). Since classifiers use a multivariate approach, and therefore discriminations are based on the global spatial pattern, local inferences should never be made in regards to the weights. For each classifier, in line with Mourao-Miranda et al. (16), we set a threshold of 30% of the maximum positive and negative weight values to generate a spatial representation of the regions that most contributed to the group discrimination. These maps are illustrated in Figure 2.

### Statistical analysis

We used four different measures – SVM based decision values, DOR, NNP, and Cohen’s Kappa – to compare the 7- and 3-T GM and WM classifiers.

Support vector machines use decision values (the distance of a data point from the hyperplane) when computing the optimal margin between groups. Decision values represent the confidence an SVM classifier has in its decision regarding the group (or class) membership of a datapoint. Higher absolute value for a data point suggests that the classifier has a high degree of confidence in its decision. Comparison of decision values provides a quantitative evaluation of the confidence of each classification made by the classifier (24). We quantified the variation in the performance of classifiers in patients by comparing decision values across different field strengths (3/7 T) and tissue types (GM/WM) using paired *t*-tests.

Diagnostic odds ratio and NNP were calculated as measures of diagnostic utility for GM and WM classifiers at both 7 and 3 T strength. DOR provides a ratio of the odds of positivity relative to negativity in disease relative to the odds of positivity relative to negativity in the non-diseased, written as:

$$\text{DOR}=\frac{\text{TP}}{\text{FN}}/\frac{\text{FP}}{\text{TN}}=\frac{\text{sensitivity}}{\left(\text{1}-\text{sensitivity}\right)}/\frac{\left(\text{1}-\text{specificity}\right)}{\text{specificity}}$$

where TP, FP, FN, and TN represent true positive, false positive, false negative, and true negative respectively (27). A value of one indicates inability for the classifier to discriminate between patients with a disease and healthy controls. The higher the value, the more superior is the discriminatory performance. The NNP is derived from the predictive summary index (PSI), which provides a measure of gain in certainty when a diagnostic tool is used in a target population (26). PSI is computed as PSI = (PPV + NPV − 1), where PPV refers to positive predictive value (or post-test probability of the diagnosis = the proportion of positive results that are correct) and NPV refers to negative predictive value (or post-test probability of the absence of diagnosis = the proportion of negative results that are correct). PPV and NPV are Bayesian measures of gain in conditional certainty.

For the test positive condition, the gain in the certainty for the diagnosis is the difference between the post-test probability for the diagnosis (the PPV) and the prior probability (the prevalence). In other words, prevalence (PPV) gives the gain in certainty for positive test. For the test negative condition, the gain in the certainty for the absence of diagnosis is the difference between post-test probability of absent diagnosis (the NPV) and the prior probability of no disease, i.e. (1 − PPV). In other words, [NPV − (1 − prevalence)] gives the gain in certainty for a negative test. The total gain in certainty (termed as PSI) then is the addition of these two.

$$\text{PSI}=\text{PPV}-\text{prevalence}+\left[\text{NPV}-\left(1-\text{prevalence}\right)\right].$$

This can be rewritten as

PSI=PPV+NPV−1

The NNP is an estimate of the number of patients that need to be examined in order to correctly predict diagnosis in one person. This can be calculated using Linn and Grunau (26) formula: NNP = (1/PSI).

Two distinct classifiers need not always classify the *same* individuals as patients, despite both having a high overall diagnostic accuracy. We quantified the degree of agreement between the classifiers using Cohen’s kappa (*K*).

## Results

### Classifier performance

The 3-T classifier discriminated schizophrenia cases and controls with 66.6% accuracy (70% specificity; 63.2% sensitivity) for GM and 63.9% accuracy (70% specificity; 57.9% sensitivity) for WM. The 7-T classifier performed at a slightly higher accuracy rate of 77% (75% specificity, 78.9 sensitivity) for GM and 69.1% (75% specificity, 63.2% sensitivity) for WM. These results are shown in Table 2 and all test performances were significantly better than chance.

**Table 2:** **Results of the 3- and 7-T classifiers (*p* values in parentheses)**.

	Balanced accuracy (*p*)	Specificity (*p*)	Sensitivity (*p*)	Positive predictive value (PPV)	Negative predictive value (NPV)	Mean decision value (patients only) (SD)	PSI	NNP	DOR
3 T									
GM	66.6 (0.018)	70 (0.026)	63.2 (0.017)	66.7	66.7	−0.004 (0.249)	0.334	2.994	4.004
WM	63.9 (0.022)	70 (0.024)	57.9 (0.036)	64.7	63.6	0.006 (0.247)	0.283	3.53	3.211
7 T
GM	77 (0.001)	75 (0.005)	78.9 (0.001)	75	78.9	−0.24 (0.447)	0.539	1.855	11.23
WM	69.1 (0.013)	75 (0.011)	63.2 (0.028)	70.6	68.2	−0.211 (0.465)	0.388	2.577	6.005

A paired *t*-test of the decision values of the patients showed a significant difference between the 3- and 7-T GM classifiers [*t*(18) = 2.435, *p* = 0.026] and also between the 3- and 7-T WM classifiers [*t*(18) = 2.137, *p* = 0.047]. No significant differences were seen between tissue type for either 3 or 7 T classifiers. Results are displayed in Table 3.

**Table 3:** **Results of paired *t*-test of decision values of schizophrenia patients (*N* = 19)**.

	Paired *t*-test	
	*T* score	Significance
3 T GM vs. 3 T WM	0.528	*p* = 0.604
7 T GM vs. 7 T WM	1.086	*p* = 0.292
7 T GM vs. 3 T GM	2.435	***p* = 0.026**
7 T WM vs. 3 T WM	2.137	***p* = 0.047**

*Bold p values significant at p < 0.05*.

The DOR demonstrated superiority of both the 7-T classifiers relative the respective 3 T classifiers. In addition, the scores for both 3 and 7 T classifiers indicate GM to be superior to WM for classifying controls from patients. The 3-T classifier scored 4.004 and 3.211 for GM and WM respectively, while the 7-T classifier scored 11.23 and 6.005 for GM and WM respectively. The PSI and NNP suggested better performance for the 7-T GM classifier, whereby two (1.855) patients would be required to be examined to correctly diagnose an individual with disease using the 7-T GM analysis, compared to three or more patients required for any other classifier (3 T GM: 2.994; 3 T WM: 3.53; 7 T WM: 2.577).

Cohen’s kappa resulted in agreement of 0.59 between 3 and 7 T classifiers for both GM and 0.49 for WM, indicating moderate agreement in classification. The 7-T model (both GM and WM) correctly classified an additional six individuals that were incorrectly classified by the 3-T classifier. There was high level of agreement between tissue type for both the 3- and 7-T (*K* = 0.74 and 0.85, respectively) classifiers.

## Discussion

We have demonstrated that the magnetic field strength of a scanner and the tissue type chosen for morphometric analysis can have a substantial impact on the performance of neuroanatomical pattern classifiers discriminating patients with schizophrenia from healthy controls. Accuracy, specificity, and sensitivity were all improved by the use of a higher magnetic field strength. The incremental benefit from the superior diagnostic ability of the 7-T GM “test” can obviate the need for 1 additional test for each correct diagnosis when compared to “tests” at lower scanner strength or employing WM features.

We have shown that the pathological changes observed in both the WM and GM tissues are of significant discriminatory value for separating patients with schizophrenia from healthy controls. The accuracy levels observed in the present study are comparable to several previous studies [(14, 16, 17); e.g., Ref. (4, 23)], while others have achieved better performance [(15); e.g., Ref. (8, 20, 21)]. Most of the pattern classification studies in psychosis have focused on GM features. Relatively few studies that consider WM features in addition to GM highlight the presence of discriminatory features in WM as well (14, 23). For the first time we have compared head-to-head the incremental benefits of the two tissues in SVM analysis. Our results suggest that tissue type chosen for morphometry alone is unlikely to have a significant influence on the classification accuracy when identifying patients with schizophrenia from healthy controls. This is also apparent when considering Cohen’s kappa measures between GM and WM classifiers. This is not surprising, given that diffuse abnormalities affecting both GM and WM define the neuroanatomical landscape of schizophrenia. On the other hand, combining the diagnostic information from the two modalities may offer specific advantages. For example, if both 7 T GM and WM classifiers are administered simultaneously to a target population (with 70% PPV of schizophrenia) and if only those subjects who are classified to belong to the patient group by both classifiers are offered the diagnosis of schizophrenia, the post-test probability of the “combined” positive test in this scenario will rise to 94.9% (assuming that GM and WM are truly “independent” tests). It is imperative that future studies test such assumptions and scenarios in real-world practice to guide clinical protocols.

For the first time in SVM studies in psychosis, we report the DOR, a single indicator of test performance that is not influenced by variations in disease PPV. This index facilitates meta-analysis of diagnostic studies and will greatly aid in consolidation of evidence from various SVM studies published so far. Being invariant to the variations in the population characteristics such as the PPV, DOR offers a useful index for comparing classifier performance reported in a variety of clinical settings. Routine reporting of this index will allow an unbiased estimation of the classification and enable the process of selecting an optimum test for specific clinical applications.

Whilst odds ratios are intuitive and allow a clinician to compare various tests, they do not provide an immediate assessment of the impact of a test on health-care costs. Estimating the number need to predict (NNP) reflects the collective benefit to patients when a test enters routine clinical use. NNP is comparable to measures such as number needed to treat (NNT) and number needed to harm (NNH) that are commonly used in clinical settings to provide easily understood measures of relative efficacy of different treatments. Such measures are widely used in clinical decision-making, and in evidence based clinical practice. They also facilitate meaningful cost-effectiveness analysis for health economic evaluation.

**Figure 3:**
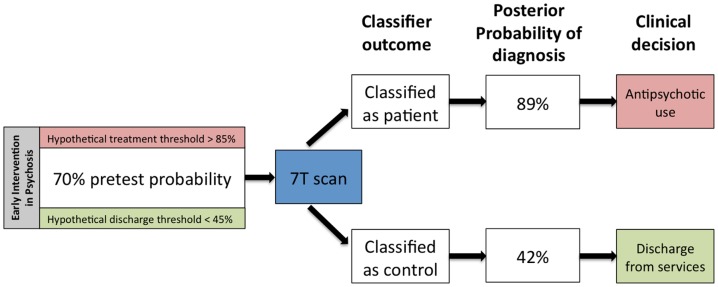
**Hypothetical changes in clinical practice that can be expected if the 7-T gray matter classifier reported in this study enters clinical use**. Given the prevalence of a diagnosable psychotic disorder to be 70% in an early intervention service, a classification outcome favoring the presence of diagnosis can tilt the balance toward antipsychotic prescription, while an outcome favoring the absence of diagnosis can aid in making a decision of discharge from services. This example assumes two hypothetical decision thresholds: one at 85% diagnostic confidence for antipsychotic prescription and the other at 45% diagnostic confidence to discharge a patient from early intervention services.

Cohen’s kappa measures used in the present study indicated only moderate levels of agreement among the 7- and 3-T classifiers. For example, the 7-T GM classifier was able to correctly classify six individuals who could not be correctly classified by the 3-T GM classifier. This finding is of crucial importance for SVM studies in psychosis. So far, most individual classifiers have been observed to achieve accuracy levels of 70–80%, which is well below the 90% mark that may be required for routine clinical use of MRI (14). The presence of a moderate rather than a high degree of agreement between two tests suggests that the results are not entirely dependent, and additional information could be gained by combining the two tests. Bayesian approaches to combine diagnostic tests have been applied in other fields of medicine where gold-standard diagnostic procedures are lacking (34), and have a high degree of appeal in translational neuroimaging in psychosis. If we consider *a priori* probability of 70% confidence for true diagnosis of psychosis (35) and apply Bayesian theorem, the use of the 7-T GM SVM classifier to identify schizophrenia would increase the likelihood for a positive diagnosis to 89%, whereas a negative finding would reduce the likelihood to 42% (Figure 3). Hypothetically, these probabilities may be used as thresholds to determine whether an individual undergoes treatment or be discharged. For example, medication would be initiated in those that reached probability of 80%, while those with probability of 45% would be discharged. Therefore, SVM can provide useful additional information toward diagnostic decisions, especially for inconclusive cases. This warrants similar Bayesian approaches across various modalities in order to determine the variables that would enable maximal additional certainty toward diagnosis. In terms of the current study, we acknowledge that sequential 7 and 3 T scans may not have a place in routine clinical care. However it is important that future studies investigate the degree of covariance (or conditional dependence) between neuroimaging modalities that can be obtained in a single session (e.g., resting fMRI and structural MRI, or DTI and WM morphometry), with a view to investigate the degree of additional certainty that might be gained by using automated classification algorithms.

**Figure 4:**
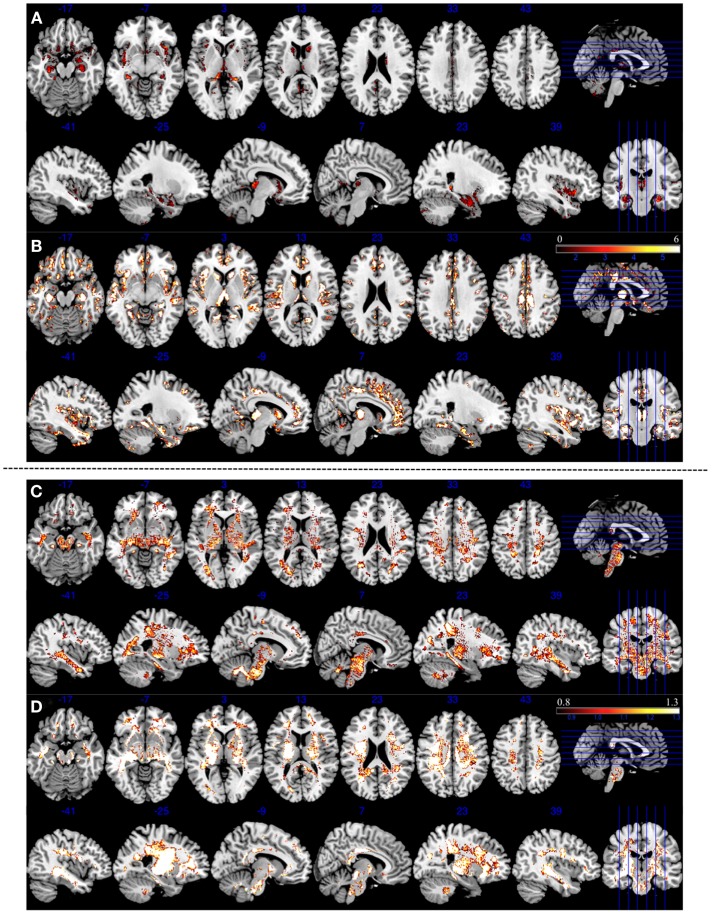
**Areas of reduced gray and white matter volume in patients with schizophrenia compared to controls at two different field strengths (3 and 7 T)**. Top panel **(A,B)** represents gray matter morphometry. Bottom panel **(C,D)** represents white matter morphometry. All contrasts are based on unsmoothed tissue segmentations, reported with uncorrected *p* = 0.1 threshold to enable visualization of localized morphometric changes. **(A)** Three Tesla GM contrasts showing reduced bilateral insula, parahippocampal, and thalamic volume reduction. **(B)** Seven Tesla GM contrasts showing more widespread volume reduction involving bilateral insula, parahippocampal region, thalamus, anterior cingulate cortex, and superior temporal regions. **(C)** Three Tesla WM revealing widespread WM changes, spatially coinciding albeit less pronounced in intensity when compared to 7 T WM changes shown in **(D)**. Color bars represent *T* values for GM and WM contrasts in both 3 and 7 T field strengths.

A number of previous VBM studies consistently reveal GM reduction in the insula, anterior cingulate cortex, thalamus and parahippocampal gyrus (36–39), and volumetric (40) or structural integrity changes measured using anisotropy (41) in the WM in several fronto-temporal regions in schizophrenia. Although we cannot make localized inferences regarding the brain regions contributing to classification using a SVM approach (32), undertaking a VBM analysis in the same dataset can reveal the brain regions showing maximal changes in patients compared to controls. Given the modest power of our sample to detect VBM differences in the unsmoothed data that entered SVM analysis, we generated controls vs. patients GM contrast at an uncorrected threshold of *p* = 0.1 to compare the consistency between 3 and 7 T in detecting regional differences. We observed GM reduction in bilateral insula, anterior cingulate cortex, thalamus, superior temporal cortex, and parahippocampal gyrus at both 3 and 7 T. But these differences were far more pronounced at 7 T than 3 T. A similar pattern of more pronounced findings at 7 T compared to 3 T was also observed for voxel based WM deficits (Figure 4).

There are several limitations to be considered when interpreting current findings. We had a relatively small sample with back-to-back scans in the current study. As the discriminatory ability of the classifiers may depend, to some extent, on the size of the training dataset, it is possible that the difference between 7 and 3 T classifiers may become weaker (or stronger) in larger samples. Abdulkadir et al. (24), investigated this issue in Alzheimer’s disease and reported that contrary to expectations, smaller training sets are not more vulnerable to changes in the MR hardware. Importantly, the use of a larger training set did not decrease decision errors. This suggests that the incremental gain observed for 7 T GM classifier should persist in larger samples as well. In the current study, we were limited to the comparison of two features used in SVM studies of psychosis – WM and GM morphometric measures from structural MRI. We did not have data to compare other commonly used modalities such diffusion tensor imaging of WM which appear promising (7, 12) or functional MRI. A comprehensive evaluation to establish both the incremental validity and combinatorial utility of the various imaging modalities is crucial for further progress of translational neuroimaging in psychosis. Such approaches should not be limited to the comparison of patients and highly selected healthy controls, but must be extended to epidemiological neuroimaging data involving a wider spectrum of individuals including high-risk subjects and patients with different prognostic outcomes. The acceptability of a diagnostic procedure is a central feature contributing to its clinical effectiveness. Seven Tesla MR scanner has a higher likelihood of producing transient light-headedness and dizziness due to the higher strength of the magnetic field (42). In the current study, we did not have any subjects discontinuing the study due to side effects. Nevertheless, the possibility of poor tolerance in some subjects must be borne in mind when considering wider use of 7 T. We also note that 7 T scanners are not widely available; this calls for further studies to systematically evaluate the utility of pattern classification framework using more widely available imaging approaches. Such studies will be essential to determine the most pragmatic high performance tool (or tools) for clinical use.

In summary, the present study has highlighted the need for systematic assessment of factors influencing the performance of pattern classification approaches in psychiatric neuroimaging. We have also evaluated clinically intuitive measures that can be used in the comparison of various classifiers. DOR provides a mean for synthesizing SVM based findings in a meta-analytical framework, which will be crucial in aiding clinical translation. NNP provides a summary value for measuring economic effects and resource allocation required to deploy SVM based diagnostic tools at service level. For individual patients, predictive values offer directly useful numerical measurement of the likelihood of a diagnostic outcome that can guide treatment decisions, as highlighted in our hypothetical example. This is an initial step toward translating psychiatric neuroimaging from ‘the scanners to the services.”

## Conflict of Interest Statement

Lena Palaniyappan has received a Young Investigator Fellowship sponsored by Eli Lilly in 2010; receives book royalties from Oxford University Press (UK). Peter F. Liddle received honoraria for an academic meeting from Bristol-Myers Squibb in the last 3 years. Sarina Iwabuchi reports no potential conflicts of interest in relation to this work.
